# Development of an integrated, district-wide approach to pre-pregnancy management for women with pre-existing diabetes in a multi-ethnic population

**DOI:** 10.1186/s12884-018-2028-2

**Published:** 2018-10-15

**Authors:** Maryam Sina, Freya MacMillan, Tinashe Dune, Navodya Balasuriya, Nouran Khouri, Ngan Nguyen, Vasyngpong Jongvisal, Xiang Hui Lay, David Simmons

**Affiliations:** Western Sydney University, Sydney, NSW 2751 Australia

**Keywords:** Pre-pregnancy care, Contraception, Type 1 diabetes, Type 2 diabetes, Intervention programs, Malformations

## Abstract

**Background:**

Poor diabetes management prior to conception, results in increased rates of fetal malformations and other adverse pregnancy outcomes. We describe the development of an integrated, pre-pregnancy management strategy to improve pregnancy outcomes among women of reproductive age with diabetes in a multi-ethnic district.

**Methods:**

The strategy included (i) a narrative literature review of contraception and pre-pregnancy interventions for women with diabetes and development of a draft plan; (ii) a chart review of pregnancy outcomes (e.g. congenital malformations, neonatal hypoglycaemia and caesarean sections) among women with type 1 diabetes (T1D) (*n* = 53) and type 2 diabetes (T2D) (*n* = 46) between 2010 and 2015 (iii) interview surveys of women with T1D and T2D (*n* = 15), and local health care professionals (*n* = 13); (iv) two focus groups (*n* = 4) and one-to-one interviews with women with T1D and T2D from an Australian background (*n* = 5), women with T2D from cultural and linguistically diverse (CALD) (*n* = 7) and indigenous backgrounds (*n* = 1) and partners of CALD women (*n* = 3); and (v) two group meetings, one comprising predominantly primary care, and another comprising district-wide multidisciplinary inter-sectoral professionals, where components of the intervention strategy were finalised using a Delphi approach for development of the final plan.

**Results:**

Our literature review showed that a range of interventions, particularly multifaceted educational programs for women and healthcare professionals, significantly increased contraception uptake, and reduced adverse outcomes of pregnancy (e.g. malformations and stillbirth). Our chart-review showed that local rates of adverse pregnancy outcomes were similarly poor among women with both T1D and T2D (e.g. major congenital malformations [9.1% vs 8.9%] and macrosomia [34.7% vs 24.4%]). Challenges included lack of knowledge among women and healthcare professionals relating to diabetes management and limited access to specialist pre-pregnancy care. Group meetings led to a consensus to develop a district-wide approach including healthcare professional and patient education and a structured approach to identification and optimisation of self-management, including contraception, in women of reproductive age with diabetes.

**Conclusions:**

Sufficient evidence exists for consensus on a district-wide strategy to improve pre-pregnancy management among women with pre-existing diabetes.

**Electronic supplementary material:**

The online version of this article (10.1186/s12884-018-2028-2) contains supplementary material, which is available to authorized users.

## Background

Women with type 1 and type 2 diabetes have a high risk of adverse pregnancy outcomes, especially when conception is unplanned [1]. Uncontrolled hyperglycaemia before and during pregnancy leads to adverse maternal and foetal outcomes [2]. Pre-pregnancy care (PPC) includes services aimed at managing glycaemia, education on diabetes complications in pregnancy, screening and treatment of diabetic complications, management of medications, and the supplementation of higher-dose folic acid required by women of reproductive age to ensure higher chances of an uncomplicated pregnancy [3]. A meta-analysis in 2012 showed that across 12 studies, PPC was effective in reducing congenital malformation [Risk Ratio (RR) = 0.3 (95% CI 0.2, 0.4)] and perinatal mortality [RR = 0.3 (95% CI 0.2, 0.8)] [3]. PPC was associated with a lower HbA1c in the first trimester of pregnancy by an average of 1.9% (95% CI: − 2.1, − 1.8) or 20.8 mmol/mol (95% CI: − 23.0, − 19.7) [3].

However, these studies showing reduced malformations and perinatal mortality have come from clinics established for women who are planning pregnancy, and are willing and able to attend [4, 5]. Lack of information and practical constraints, such as commitment to work, are often major obstacles for those who do not attend pre-pregnancy clinics [6]. Furthermore, approximately 50% of pregnancies among women with pre-existing diabetes remain unplanned [7, 8], and many of these should therefore be seen as largely a failure of contraception rather than a failure of access to a pre-pregnancy clinic. However, a proportion of these ‘unplanned’ pregnancies occur among women who wish to maintain autonomy during the pregnancy process [9], and who would not attend a pre-pregnancy clinic. It is clear that a pregnancy in a woman with diabetes without PPC is often a failure of ‘the system’, although at times this may be due to the failure of clinicians to understand the perspective of women with diabetes. In fact, the term “PPC” could be misleading as it implies that women are planning pregnancy, when all that may be required is effective contraception or knowledge about the options and methods of their use. Therefore, we use the term ‘pre-pregnancy management’ (PPM), to emphasize the integrated approach required to improve contraception uptake and pregnancy outcomes in this population.

There have been a variety of interventions at a population level that could be used for PPM, including better contraception choices, in order to reach women who have not traditionally attended pre-pregnancy clinics [5, 10–13]. However, no population-based intervention program has been implemented in Australia. South West Sydney (SWS) is a home to women from culturally and linguistically diverse (CALD) backgrounds who may have different risks to women participating in previous population based approaches, requiring additional or different PPM [1, 14]. We now present the steps in the development of an intervention program for PPM, including enhanced contraception uptake, for the multiethnic district of SWS.

## Methods

The development of the intervention involved: 1) a narrative literature review of PPC intervention programs used in women with type 1 diabetes (T1D) and type 2 diabetes (T2D); 2) chart review of women with T1D and T2D; 3) interview surveys with women with T1D and T2D and health care professionals (HCPs); 4) in-depth qualitative exploration of local pregnancies through focus groups one-to-one interviews with women with T1D and T2D and partners; 5) the preparation of a draft intervention plan with discussion at a local conference; and 6) a discussion of the intervention at two multidisciplinary meetings one, predominantly primary care (*n* = 15) and one predominantly secondary care (e.g. general practitioners, obstetricians, midwives, endocrinologists, diabetes educators, dietitians) (*n* = 17) to finalise the plan.

### Narrative review

**Fig. 1:**
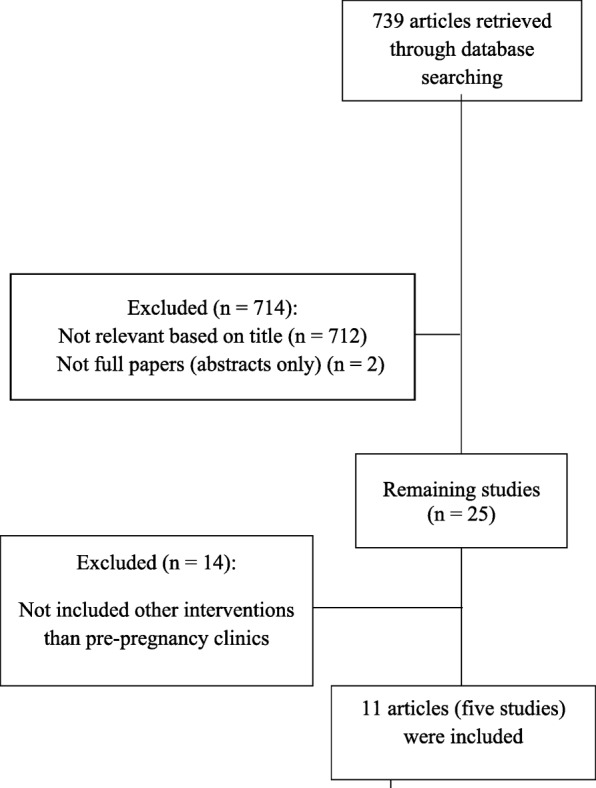
Process of selection of the studies for the literature review

Google Scholar, PubMed and Medline databases were searched for randomised trials (including cluster and quasi-randomised trials), as well as cohort and case-control studies assessing the impact of PPC programs on women’s knowledge of the components of PPC and the use of contraception and/or their pregnancy outcomes (Fig. 1). Studies investigating solely the effect of (a) pre-pregnancy clinics on pregnancy outcomes, as opposed to the effect of other interventions (e.g. leaflets, posters) were not included [3]. The search strategy included the following keywords: “pre-conception care” or “intervention programs” or “contraception” or “contraception uptake” or “pre-pregnancy care” AND “negative pregnancy outcomes” or “adverse pregnancy outcomes” or “pregnancy complications” AND “type 2 diabetes” or “type 1 diabetes” or “diabetes”. Only full-text journal articles in English of studies that assessed the impact of PPC programs on women’s knowledge and/or their pregnancy outcomes were included. Additional information was obtained from authors of publications where required.

### Chart review

Hospital records (in electronic and paper formats) of pregnant women with pre-gestational T1D (*n* = 53) and T2D (*n* = 46) from Macarthur Diabetes in Pregnancy Clinic (MDPC), between 2010 and 2015, were reviewed. To minimise classification bias, medical records were re-reviewed. Data that were extracted from the charts included parity, age and body mass index (BMI), type of diabetes (type 1 vs. type 2 vs other), family history of diabetes, type of treatment (e.g. insulin, Metformin) at conception and during pregnancy, folate use, country of birth, third trimester HbA1c (%), mode of delivery (e.g. vaginal, caesarean section, vacuum and forceps), as well as pregnancy outcomes including neonatal hypoglycaemia, congenital malformations [15], macrosomia (birth-weight ≥ 4000 g or > 90% centile) [16], stillbirth, pre-eclampsia and hypertension (blood pressure ≥ 140/90 mmHg) [16].

### Interview surveys

Fifteen women and 13 HCPs were asked about their current and previous experiences of pre-pregnancy diabetes care/management and for suggestions to improve care, using closed and open questions either face to face or over the telephone. HCPs included General Practitioners (GPs) (*n* = 2), endocrinologists (*n* = 3, private and ‘public’), gynaecologists (*n* = 3), diabetes educators (*n* = 2), a dietitian (*n* = 1) and midwives (*n* = 2). Answers were summarised in relation to two identified over-arching key themes (contraception and PPM).

### Focus groups and one to one interviews

Two separate focus groups were conducted involving two women with T1D, another including two women from CALD backgrounds (Muslim and Asian). Due to lack of flexibility in time for women and their partners to attend the focus-group sessions, 12 in-depth one-to-one interviews were also conducted using the same questions with women from an Australian background with T1D (*n* = 2) and T2D (*n* = 1), women with T2D of CALD background (*n* = 5) as well as partners of CALD women with T2D (*n* = 3), and an Aboriginal woman with T2D (*n* = 1). Each focus group/interview was audio recorded and transcribed verbatim.

### Development of a PPM plan

The development of a draft PPM intervention plan was based on the identified approaches from the narrative review, using the components of previous studies (e.g. East Anglia Study group for Improving Pregnancy Outcomes in women with Diabetes (EASIPOD) [5]) and was then amended addressing gaps identified from the results of the chart-review, interview surveys and focus groups/interviews (Table 3). The plan components were shared in a local conference comprising multi-disciplinary HCPs and researchers. The attendees voted for each component based on feasibility, practicality and sustainability. The draft proposal was then discussed in two face-to-face meetings using a Delphi approach [17, 18]. The first focussed on the perspective from primary care, including GPs (*n* = 12); a dietitian (*n* = 1) and endocrinologists (*n* = 2). Each proposed PPM/contraception component was evaluated using a pros and cons list.

**Fig. 2:**
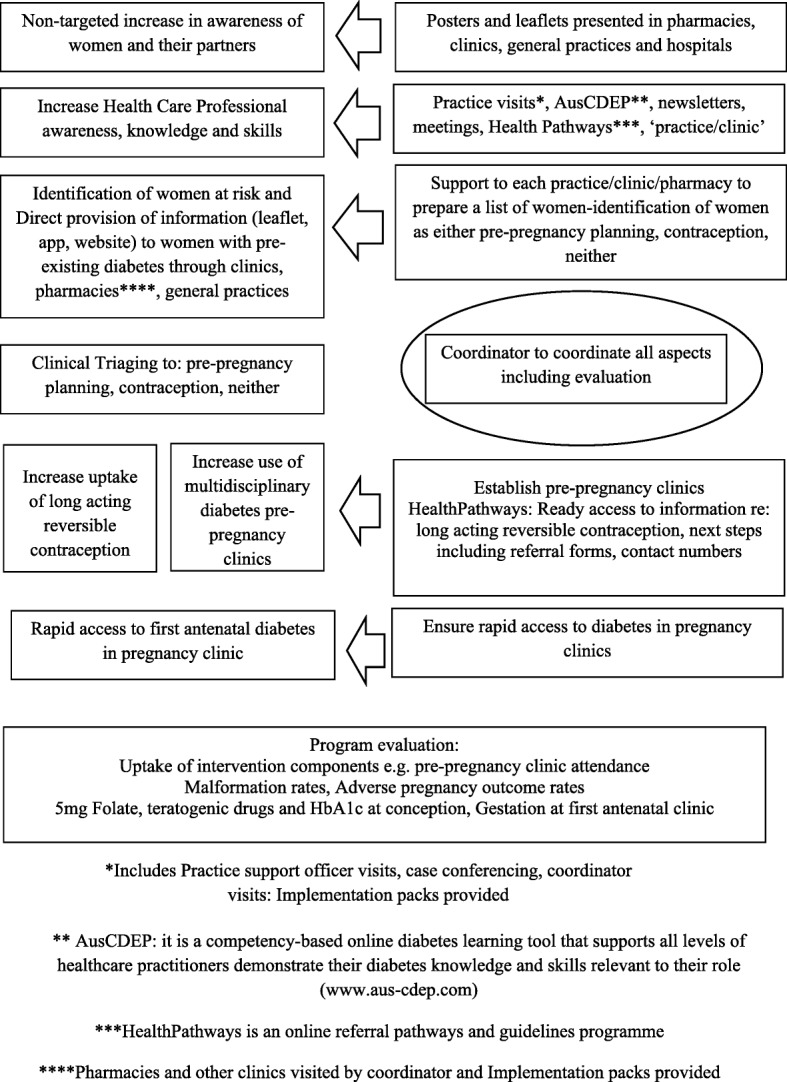
The process of implementing the Diabetes Contraception and Pre-pregnancy Program

The final meeting involved key stakeholders from across the district (each of the 5 hospital facilities and primary care) representing a range of views from endocrinologists (*n* = 4), a diabetes educator, midwives (*n* = 5), obstetricians/gynaecologists (*n* = 2), a GP, a dietician, a clinical manager and health economists (*n* = 2). The meeting was facilitated by an endocrinologist. Those present formed three groups: two hospital facility groups and ‘the rest’ to discuss possible components of the intervention that related to their facility/perspective (Fig. 2). Once all groups reached consensus for each component of the program, responses were debated across the meeting, creating an opportunity for open discussion and combining or modifying the group outcomes. The final plan arose from this meeting.

### Analyses

In the chart review, demographic characteristics and pregnancy outcomes among women with T1D and T2D were compared using chi-squared (categorical data) and *t*-tests (numerical data). Multivariate analyses (logistic regression and linear regression) were performed to adjust for age to compare the differences in characteristics between women with T1D and T2D. All analyses were performed using Stata v14. All tests were 2-tailed and significance was taken as *P* < 0.05.

Qualitative data from the open-ended interview survey questions, focus groups and individual interviews were thematically analysed identifying topics and substantive categories within participants’ accounts in relation to the study objectives. This helped to identify topical responses and emergent substantive categories, coding particularly for word repetition, direct and emotional statements and discourse markers including intensifiers, connectives and evaluative clauses. Responses and quotes from individuals participating in focus groups and interviews and responding to open ended questions from the interview surveys, are shown by alphanumeric coding, where women are labelled ‘W’ and men ‘M’ and the type of diabetes is indicated (e.g. W102- T1D = participant 102 who was a woman with type 1 diabetes).

## Results

### Narrative review

Figure 1 shows the number of studies identified and reasons for exclusion in the narrative review. As shown, from 739 studies, 714 studies were excluded in the first screening phase. One further publication emerged following the review [19]. As shown in Table 1, five studies (published in 11 articles), which assessed different aspects of each type of intervention, were identified. Of the five studies, four used multi-faceted approaches (e.g. books, CD-ROM, posters, leaflets, HCP educational courses, counselling sessions, online learning materials for HCPs) [5, 9, 12, 19–22] and one used a mono-faceted approach (DVDs) [10, 13, 23, 24]. All approaches contained information on the importance of pregnancy planning, use of contraception, maternal and baby risk, and PPM components [10, 13, 19, 23, 24].

**Table 1:** Literature-review of pre-pregnancy programs

Studies and interventions	Region/ Country	Program content	Specialist delivered the interventions	Centres	Results
DVD [10, 13, 23, 24]	Northern Island/UK	The importance of planning pregnancy and the role of contraception.	A diabetes specialist- nurse and -midwife, a dietitian, a GP, a clinical health Psychologist, an obstetrician, a nutritionist	**1** [10]: Two national health services **2** [13]:At their centre **3** [23]**:** Five diabetes care within the five health and social care trust and general practices; **4** [24]: At their centre	**1** [10]: The DVD significantly improved self-efficacy and reduced perceived barriers. Knowledge of pregnancy planning and pregnancy-related risks increased (*P* < 0.001). **2** [13]: The development process and outcome evaluation are an important point of reference for future educational programs **3** [23]: The viewed-DVD subgroup had lower first visit HbA1c (*P* < 0.001; increased planned pregnancy (*P* < 0.001); increased folic acid preconception (*P* = 0.001); and had improved HbA1c preconception (*P* < 0.001). **4** [24]: the development of an e-learning continuing professional development resource within the website.
EASIPOD^a^ [5, 9, 19]: websites, workshops for HCP, Leaflets, structured audit with benchmarking, poster formal and informal patient education programs	East- Anglia/ UK	Planning a pregnancy and contact details for local PPC coordinator	Diabetes physician, specialist nurse, midwife or obstetrician	Primary-care teams in community settings, women with T1D: by specialist teams in hospital settings. Joint clinics.	**5** [5]: Women with PPC presented earlier (*P* = 0.001), were more likely to take 5 mg preconception folic acid (*P* = 0.0001) and had lower HbA1c (*P* = 0.0001). They had fewer adverse pregnancy outcomes *P* = 0.009). Lack of PPC was independently associated with adverse outcome (OR = 0.2; 95% CI 0.05–0.89). **6** [9]: Understanding PPC (90%); optimal glycaemic control (80%); risks of malformation (48%) and macrosomia (35%). 70% were not regularly using contraception (70%), stopped deliberately (45%), become less rigorous (28%) or experienced side effects (14%).
EASIPOD 2^a^ [19]: websites, workshops for HCP, Leaflets, structured audit with benchmarking, poster formal and informal patient education programs; GP software flags, online education program for HCPs	East- Anglia/UK	Planning a pregnancy and contact details for local PPC coordinator	Diabetes physician, specialist nurse, midwife or obstetrician	Primary-care teams in community settings, women with T1D: by specialist teams in hospital settings. Joint clinics.	**7** [19]: In those withT1D: improved gestational age at booking (7.6 vs 8.4 weeks), and in women with T2D: high rate of first HbA1c of < 6.5% < 48 mmol (58.5% vs 44.4%) and higher rate of preconception 5 mg folic acid (41.8% vs 23.5%)
READY-Girls [12, 20, 21]	Pittsburgh/USA	Presents the effects of diabetes on reproductive health, puberty, sexuality, and pregnancy and the benefits of PPC and includes skill-building exercises for healthy decision making and communication with HCPs.	Specialised nurses and GPs	Major university-based diabetes clinics	**8** [20]: Improved knowledge about family planning and reproductive health issues. **9** [12]: Increased in knowledge after the first visit (*P* < 0.001) and being sustained for 9 months (*P* < 0.05). preconception counselling barriers decreased over time (*P* < 0.001), and intention and initiation of preconception counselling and reproductive health discussions increased (*P* < 0.001). **10** [21]: Stronger knowledge about PPC (*P* = 0.003) and seek PPC when planning a pregnancy\ (*P* = 0.02)
Leaflets and posters in out-patient waiting room [22]	Ireland	Patient education, a full medication review, assessment & treatment of diabetes-complications and thyroid status, commencement of folic acid 5 mg/d and focus on intensive glucose monitoring	Specialist and general practitioners	Antenatal care by Primary care clinicians, local endocrinologist, diabetes nurse specialist and dietitian	Attendees were more likely to take preconception folic acid (*P* < 0.001) and less likely to smoke (*P* = .03). Attendees had lower glycated haemoglobin levels (*P* < .001; third trimester HbA1c (*P* = 0.001), and their offspring had lower rates of serious adverse outcomes (*P* = 0.007)

^a^*EASIPOD*: East Anglia Study group for Improving Pregnancy Outcomes in women with Diabetes

Numbers in bold refer to different reports

Table 1 summarises the impact of four approaches on adverse pregnancy outcomes and/or knowledge of PPM. DVDs, the READY-Girls and the EASIPOD interventions improved the knowledge of women in pregnancy planning and contraception use [21] [23], and in PPM [12, 13]. Multi-faceted interventions (i.e. EASIPOD and leaflets and posters in healthcare facilities) decreased the rate of smoking [21], reduced HbA1c [5, 9, 22]) and increased 5-mg folic-acid uptake [5, 9, 22] at conception. The rates of adverse pregnancy outcomes including congenital malformations (7.3% vs. 4.3%; *P =* 0.04) [9]; (0.8% vs 5.2%; *P* = 0.04) [21] and perinatal mortality (1.8 vs. 3.7%, *P* = 0.07) [9] were also significantly reduced. A revised EASIPOD programme (EASIPOD 2), in which primary diabetes practitioners were far more engaged, in addition to secondary healthcare providers, showed an increased rate of pre-conception 5 mg folic-acid uptake (from 46 to 64%) in women with T1D as well as significant improvement of HbA1c at conception in women with T2D, with almost 60% of women reaching target (6.5% or ≤ 48 mmol/mol) [19].

### Chart-review

Table 2 shows that overall, women with diabetes were often overweight/obese, parous with a strong family history of diabetes. Women with T1D were largely (78.3%) from Australia/ of European descent, with those with T2D from more varied backgrounds. High dose folic acid uptake was low, with no difference between women with Type 1 and Type 2 diabetes. Third trimester HbA1c was relatively high and several women (9.5%) with T2D were medicating with potentially fetotoxic diabetes medications. Recorded retinal screening during pregnancy was low particularly (but non-significantly) in women with T2D. Malformation rates were high with an overall rate of 12.4%. The overall rates of other pregnancy outcomes were also high including Caesarean section rates (57.5%), hypoglycaemia (24.5%), hypertension/preeclampsia (23.4%) and macrosomia (29.8%). While there were no stillbirths, women with T1D had a high miscarriage rate (8.0%).

**Table 2:** Characteristics and pregnancy outcomes of women with T1D and T2D from the chart review

Variables	T1D	T2D	*P-*value^*^	T1D Melbourne (*n* = 107*)* [29]	Background population in NSW^d^ (2010) [28]
Age (years), mean (SD)	28.6 (5.6)	32.9 (5.2)	< 0.001	29.3 (5.3)	30.8
*n* = 99	*n* = 53	*n* = 46			
BMI^a^ (Kg/m^2^), mean (SD)^c^	25.8 (5.2)	35.4 (8.1)	< 0.001	27.3 (5.0) --	
*n* = 93	*n* = 48	*n* = 45			
Gravida, *n* (SD)^**^	2.6 (2.2)	3.1 (2.2)	0.17	–	
*n* = 98	*n* = 53	*n* = 45			
Parity, n (SD)^**^	1.0 (1.2)	1.5 (1.4)	0.09	–	
*n* = 98	*n* = 53	*n* = 45			
Country of birth, *n* (%)^**^			0.07^***^		
Australia	13 (35.1)	8 (19.1)		95 (89)	67.3%
European descent	16 (43.2)	12 (28.6)		–	–
India/Bangladesh	1 (2.7)	5 (11.9)		–	3.5%
Aboriginal	2 (5.4)	5 (11.9)		–	3.3%
Others	5 (13.5)	12 (28.6)		–	25.9%
*n* = 79	*n* = 37	*n* = 42			
Family history of diabetes, *n* (%)^**^	20 (66.7)	30 (83.3)	0.30	–	–
*n* = 66	*n* = 30	*n* = 36			
Third-trimester HbA1c^**^				–	–
%	7.0 (1.8)	6.5 (1.4)	0.4		
mmol/mol (SD)	53.0 (19.7)	47.5 (15.3)			
*n* = 76	*n* = 39	*n* = 37			
Folic acid, *n* (%)^**^			0.4^***^		
Nil	13 (36.1)	9 (30.0)			
< 5 mg	7 (19.4)	8 (26.7)			
5 mg	8 (22.2)	10 (33.3)			
Yes (dosage unknown)	8 (22.2)	3 (10.0)			
*n* = 66	*n* = 36	*n* = 30			
Treatment before pregnancy, *n* (%)^**^			< 0.001^***^		
Diet alone	0	4 (9.4)			
Tablets	0	11 (26.2)			
Metformin	0	8 (19.0)			
Gliclazide	0	3 (7.1)			
Janumet (Metformin + Sitagliptin)	0	1 (2.4)			
Insulin	51(96.2)	16 (38.1)			
Insulin + Metformin	2 (3.8)	7 (16.7)			
Nil	0	4 (9.4)			
*n* = 95	*n* = 53	*n* = 42			
Treatment during pregnancy, n (%)^**^			0.003^***^		
Insulin	38 (92.7)	32 (78.1)			
Metformin	1 (2.4)	2 (4.9)			
Insulin & metformin	1 (2.4)	5 (12.2)			
CSII^b^	1 (2.4)	0			
Insulin only at labour	0	1 (2.4)			
Total	41 (100)	40 (97.6)			
*n* = 82	*n* = 41	*n* = 41			
Retinopathy screening, n (%)^**^			0.06^***^		
Yes	18 (64.3)	9 (39.1)			
No	10 (43.5)	14 (60.9)			
*n* = 55	*n* = 29	*n* = 26			
Thyroid disease, n (%)^**^			0.8^***^		
Yes	6 (12.0)	5 (11.9)			
No	42 (84)	36 (85.7)			
*n* = 89	*n* = 48	*n* = 41			
Delivery methods^b^, n (%)^**^			0.005^***^		
Vaginal	12 (24.5)	18 (41.9)			
Elective CS^c^	17 (34.7)	13 (30.2)			
Emergency CS^c^	11 (22.5)	9 (20.9)			
Vacuum	3 (6.1)	4 (9.3)			
*n* = 87	*n* = 43	*n* = 44			
Pregnancy outcomes^ϕ^, *n* (%)^**^					
Neonatal Hypoglycaemia	9 (36.0)	3 (12.5)	0.12	–	–
*n* = 49	*n* = 25	*n* = 24			
Any congenital malformations	4 (9.1)	7 (15.6)	0.25	4 (4)	775 (0.8)
Major	4 (9.1)	4 (8.9)	0.55	–	–
Minor	0	4 (8.9)	–	–	–
*n* = 89	*n* = 44	*n* = 45			
Hypertension	11 (24.4)	10 (22.2)	0.25	2 (2)	6357 (6.7)
*n* = 90	*n* = 45	*n* = 45			
Pre-eclampsia	6 (12.2)	4 (10.3)	0.84	5 (5)	–
*n* = 88	*n* = 49	*n* = 39			
Macrosomia (birthweight> 4000 g)	17 (34.7)	11 (24.4)	0.50^ϒ^	47 (44)	–
*n* = 94	*n* = 49	*n* = 45			
Stillbirth	0	0	–	7 (7)	555 (0.6)
*n* = 88	*n* = 48	*n* = 40	–		–
Miscarriage	4 (8.0)	0		–	
*n* = 94	*n* = 50	*n* = 44			

^a^*BMI* body mass index; ^b^*CSII* continuous subcutaneous insulin infusion; ^c^*CS* Caesarean section; ^d^*NSW* New South Wales

^*^Age was included in all the statistical models;

^**^denominators vary due to missing values;

^***^Overall *P*-value

^ϕ^ those with miscarriage were excluded from the analyses (*n* = 4);

^ϒ^ adjusted for age, *BMI* and history of macrosomia

### Qualitative results (from interview surveys, focus groups and one to one interviews)

#### Patients

Early referral to the endocrinologist and clinic care (W102-T1D, W112-T2D), longer consultations and more frequent clinics (W105-T1D, W115-T1D), were highlighted as ways to improve the current service. Additional file 1 provides quotes reflecting each theme identified.Five women did not comment when asked how pre-pregnancy clinic care could be improved and a further three said no improvements were necessary.

Barriers to contraception and PPM included lack of awareness, limited motivation to change and/or a sense of information overload that reduced women’s engagement with the education being provided to them. Most women took contraceptives until they were ready to become pregnant. However, they perceived the pre-pregnancy information they were provided was not pertinent until after they fell pregnant.

Women indicated that being aware of complications and having resources to assist them in leading a healthy life was important. Google was a common source of information that women felt was reliable (W109-T2D, W111-T2D, W103-T2D). However, those who solely relied on being educated by family and friends received incorrect information.

Partner support and investment were key to engagement with healthy lifestyle choices. Male partners were interested in enhancing their knowledge on pre-pregnancy diabetes management and contraception methods.

#### HCPs

HCPs utilised resources from a variety of sources to enhance their knowledge of PPM including specialists, self-education, information from professional organisations, meetings/seminars/conferences/ journal clubs, through personal experience and training courses. According to HCPs, the most common barriers to pre-pregnancy planning was lack of education/knowledge and unawareness of PPM in women.

Furthermore, HCPs suggested that advertisement and awareness of services (including mention of the power of word of mouth, use of TV screens in GP surgeries and leaflets distributed through pharmacies), incorporation of discussion about contraception and pregnancy planning with women with diabetes of child-bearing age, positive relationships with other HCPs, after-hours services, education of women from HCPs, support from the patient’s partner, online services and more patient visits to GPs, could enhance PPM (Table 3). GPs highlighted the importance of referrals from primary to secondary care for PPM once women fall pregnant. GPs felt that the diabetes specialist services were best placed to provide the pre-pregnancy counselling.

**Table 3:** Current gaps in adherence to optimal care, possible interventions and actions required for developing the interventions in South Western Sydney based on HCPs’^a^ and women/partners inputs

Gaps (requirements)	Intervention programs used in the literature/ suggested by HCP’s/women/partners	Actions required to implement the interventions
Lack of time for women/patients to attend diabetes clinic	Websites, leaflets, contact details of local HCPs^a^ [5], social media	1) Providing after- hours clinics 2) Providing other educational resources (e.g. webpages, social media, and apps) 3) Reaching out to all patients and mailing them leaflets and information sheets on a regular basis
Lack of communication (miscommunication) between HCPs and patients	Workshops, newsletters, online learning resources,regular meetings and education programs [46]	1) Reminding HCPs about online resources and workshops 2) Adding techniques for communication to the existing learning materials
Lack of knowledge about PPM and contraception methods in women and their partners	Leaflets, posters, DVDs, PPM education programs and peer support	1) Developing a wide range of educational resources (e.g. posters, apps) 2) Increasing the accessibility of educational resources 3) Translating educational resources in most common languages
Disparities of preferences in receiving knowledge about PPM and contraception options	Use of a wide range of interventions (e.g. online resources, social media, leaflets and posters)	Raising awareness among patients and their partners about the ranges of interventions

^a^*HCPs*: Healthcare Professionals

### Development of the plan

At the conference, HCPs agreed that all proposed components were practical and sustainable and should be included (Table 3). The subsequent HCP meetings led to reduced emphasis on any specially made media (e.g. DVD’s) and GP checklist software (rather to include in existing software) due to their relatively high cost impact. Tables 4 and 5 show the items considered along with pros and cons of each component. The agreed core content for informational materials for both HCPs and women and their partners is shown in Table 6.

**Table 4:** Evaluation of interventions proposed for enhancement of Pre-Pregnancy Management (PPM) based on weighing pros and cons items

Interventions	Content/ details	Places (to be implemented)	Pros	Cons	Included
Workshops for HCPs^a^	Interpersonal techniques for communicating with other HCPs and patients (including CALD^b^ women), and PPM^c^	Primary and secondary care services	Motivational, Enhancing skills and knowledge	Lack of flexibility in time, expensive	Yes
DVD	‘Risk of unplanned pregnancy, and effective contraception methods’, ‘local support team’, ‘blood glucose targets, hypos and ketoacidosis’, ‘diet, delivery’ and ‘post-birth’	Primary and secondary care services including pharmacies	Easily accessible and convenient	High cost, not sustainable (can be lost/or scratched)	No
Web-based education program	PPM information, links to pre-existing YouTube channels in multiple languages e.g. Arabic and Vietnamese	Websites and social media	Easily accessible and convenient, no limits in content	Passive	Yes
Courses for patients and their partners	The importance of PPM (e.g. glycaemic control, smoking cessation and physical activity) and use of effective contraception	Primary and secondary care services, women’s health clinics	Motivational, they can ask questions	High cost, lack of flexibility in time	No
Posters presentation /T.V screen advertisement	The importance of PPM with information about available local services (contact details for local HCPs)	Waiting rooms of primary and secondary care services, pharmacies, women’s health and fertility clinics	Easily visible, encourage an active response	Limited content	Yes
Peer support/web chat	Sharing experiences about diabetes in pregnancy and services they have used	DCAPP social media	Easily accessible and convenient	Possibility of inaccuracy (Vulnerable to (cognitive) biases)	Yes
Text message reminders	Links to the important websites, available resources (e.g. local pre-pregnancy clinics, social media)	Will be sent from the GP practices on regular bases (every six weeks)	Easily accessible and convenient	High cost	No
Leaflets	Links to useful websites, potential risks of unplanned pregnancy and risk factors for potential complications	Primary and secondary care services, mail, pharmacies and women’s health clinics	Easy to access	High cost (if mailed), lack of interest (so common)	Yes
Apps	‘Risk of unplanned pregnancy, and effective contraception methods’, ‘local support team’, ‘blood glucose targets, hypos and ketoacidosis’, ‘diet, delivery’ and ‘post-birth’	DCAPP website and social media, leaflets, and posters	Systematic approach, no cost to design (already existed)	Only available to smart-phone users	Yes
Social media	Useful websites (e.g. NDSS^d^), updates/posts on the importance of PPM and contraception, and YouTube channel	Online (i.e. Facebook and Instagram)	High chance of being visited regularly	Only available to DCAPP social media followers	Yes
Checklist software for general practitioners	Medication review, contraception advice, weight management strategies, importance of having optimal glycaemic control	GP surgeries	Systematic approach	High cost of design Needs to articulate with existing software	No

^a^*HCPs*: health care professionals

^b^*CALD*: culturally and linguistically diverse

^c^*PPM*: pre-pregnancy management

^d^*NDSS*: National Diabetes Service Scheme: An initiative of the Australian Government administered with the assistance of Diabetes Australia [47]

**Table 5:** Interventions to increase contraception uptake- Pros and cons based on literature as well as conference and survey/focus group discussions

Interventions	Content/ details	Places (to be implemented)	Pros	Cons	Included
Workshops for HCPs^a^ and pharmacies [48]	Available contraceptive methods for women with diabetes and insertion techniques for IUD^b^	Primary and secondary care services and pharmacies	Potentially motivates HCPs, updates their knowledge	Lack of flexibility in time, High cost	Yes
Courses for patients and their partners	The importance of planning for pregnancy and available contraception options for women with diabetes	Primary and secondary care services and pharmacies	Potentially motivates patients and their partners, they can ask questions	Lack of flexibility in time, High cost	No
Accessibility of contraception	Providing free condoms in health-care services (especially primary care centres)	Primary and secondary care services, dental clinics, women’s health clinics, NDSS^c^	Easily visible, encourages people to use contraception	High cost	No
Leaflets women and their partners’ awareness [49]	Importance of planning pregnancy and contraception uptake in women with diabetes	Primary and secondary care services, women’s health clinics and NDSS^c^	Minimises potential conflicts which could exist within the couples	High cost if mailed	Yes
Mass-media, community and interpersonal channels [50]	Benefits of IUD, wide range of available contraception options, importance of optimised diabetes management prior to pregnancy	Primary and secondary care services, pharmacies and women’s health clinics	Repetitions, accessible to the majority of population group	High cost, not usable/usable for CALD women	No
Web-based program including YouTube channel	The importance of planning for pregnancy and role of contraception, education of contraception options	The app will be addressed on leaflets, posters	Accessible anytime, pre-existed	Needs internet connection	Yes
Checklist software for HCPs	Contraception advice	The link will be available on leaflet and posters	Potentially motivates HCPs, updates their knowledge	High cost Needs to articulate with existing software	No

^a^*HCPs*: Health care professionals

^b^*IUD*: intrauterine device

^c^*NDSS*: National Diabetes Service Scheme [47]- An initiative of the Australian Government administered with the assistance of Diabetes Australia

**Table 6:** Agreed core content for informational materials for both HCPs and women and their partners

Why conception with poor glucose control and/or unsafe medications should be avoided	
Contraception and family planning advice, with emphasis on the most effective contraception options (e.g. Long Acting Reversible Contraception and emergency contraception) to prevent unplanned pregnancies.	
Emphasising the importance of glycaemic control using safe medications at least three months prior to conception	
5 mg folic-acid uptake at least three months prior to pregnancy.	
Avoidance/replacement of teratogenic drugs particularly for hypertension and dyslipidaemia	
Importance of retinal, renal and vascular complication screening prior to conception	
The risk of smoking during pregnancy	
Online educational resources (e.g. National Diabetes Supply Scheme, Facebook and Instagram pages)	
Contact details of local health services	

Figure 2 shows the final components for the district-wide “Diabetes Contraception and Pre-pregnancy Program” (DCAPP) including the required resources and activities to be undertaken for the different target groups (women of childbearing age with T1D or T2D, women’s partners and HCPs). This included establishing pre-pregnancy clinics and referral pathways, disseminating educational materials relating to both planning pregnancy and avoiding unplanned pregnancies (contraception) for the woman, her partner and HCPs using a multitude of approaches including sending materials directly to the women, and establishing a programme to monitor uptake.

A key resource is the coordinator (may be more than one person), who will be appointed to oversee all aspects of the program including the educational program, data management and identifying/developing strategies to address ‘hard to reach’ women. The coordinator will additionally visit hospital clinics, general practices, pharmacies and relevant clinics (e.g. fertility clinics), to deliver and discuss implementation packs tailored to each of the four settings. General practices will also be informed of the DCAPP through visits by the local Primary Health Network Practice Support Officers and local case-conferencing (where an endocrinologist visits practices to advise on the care of individual patients with diabetes). Pharmacies will also be visited by the DCAPP coordinator and medical students.

Implementation packs include asking HCPs at each venue to, display posters, have leaflets available, show DCAPP information on TV screens, if available, and undertake brief online training about pre-pregnancy planning (AusCDEP – a competency based online multiple choice based training tool). Practice and clinic staff will be given information on how to access HealthPathways (the local online referral and clinical guideline portal) for diabetes contraception/pre-pregnancy information, patient/partner printable materials and referral advice. All staff will be asked to explain the DCAPP (briefly), offer a DCAPP leaflet to each woman of reproductive age with diabetes and ask the woman to enrol (by text, email, DCAPP website and Facebook or Instagram) to access further materials, for an annual DCAPP update and to receive any new information should it arise. How this occurs will vary by setting:

- Pharmacists will be asked to approach those picking up diabetes prescriptions.
- GP surgeries, private fertility and public diabetes clinics will be requested to make a list of the women with T1D/T2D of reproductive age and provide the coordinator with this number. This may be facilitated by using practice software. The hospital clinics have been provided with BIOGRID database/software [25] to facilitate this process. A clinic/practice member will be identified as the contact person to provide/receive further information. General practice and diabetes clinic staff who see the women will be asked to record if a leaflet has been provided, and their assessment of whether the woman is
- planning to become pregnant (and therefore warrant pre-pregnancy management/referral to the pre-pregnancy clinics or private care)
- not planning to become pregnant and identify the form of contraception in place including abstinence or not required (e.g. hysterectomy, confirmed menopause)
- neither and listing reason including informed decision, religious reasons, not currently sexually active.

The contact person will be asked to maintain an internal register of leaflets provision, pre-pregnancy and contraception status and to provide a summary to the coordinator on a regular basis (quarterly). After the first round of approaches, the assessment would occur at each annual review (unless status changes beforehand).

All HCPs will also be invited to workshops/presentations run by their organisation/professional groups to allow further dissemination and discussion about the DCAPP (One of these has already occurred with 112 general practitioners attending).

### *DCAPP Evaluation* [26, 27]

Evaluation will include assessment of uptake of the various components, qualitative evaluation of the perspectives of women, their partners and HCPs, a health economic evaluation and a comparison of pregnancy preparation and outcome measures over the first 12–24 months with those from the prior 6 years.

Program uptake will be evaluated by detailed monitoring of HCP education (AusCDEP uptake, workshop attendance, case conferencing patients), number of HealthPathways visits/clicks, number of social media followers, leaflets sent, pharmacy registrations, practice/pharmacy/clinic participation, practice/clinic reports (leaflet distribution, assessment status and long acting reversible contraception uptake reports), pre-pregnancy clinic attendance and gestation (weeks) at attendance of antenatal clinic once pregnant.

Measures of pregnancy preparation will be assessed from antenatal clinic records including: 5 mg folic acid uptake from at least 3-months preconception, whether this is a planned pregnancy or otherwise, use of potentially teratogenic medications at conception and gestational age at first visit. Other measures include first visit blood pressure, HbA1c, BMI and smoking status. Third trimester HbA1c will also be assessed.

Pregnancy outcomes will be collected from birth records including major and minor congenital malformations, stillbirths, neonatal trauma, emergency caesarean section, preeclampsia, prenatal mortality, miscarriage, preterm birth, small and large for gestational age, severe maternal hypoglycaemia, and gestational weight-gain [26, 27].

Process evaluation will include assessment of reach and the use of the DCAPP materials, through interviews in a purposeful sample of women of reproductive age with diabetes who have become pregnant (planned and unplanned) and received the materials, plus partners and HCPs. Interviews will include evaluation of the most effective mix of approaches (e.g. online and/or through pharmacies, hospital clinics and/or GP surgeries) for contacting and enrolling patients, identification of barriers and facilitators to implementation for each program component and aspects that both stimulate and obstruct use of DCAPP. Interviews will be repeated over time to facilitate continuous evaluation and quality management of the program.

Cost-effectiveness analysis will be conducted using the total intervention cost, summation of the different components, and benefits to New South Wales Health based on primary and secondary outcomes and their unit costs. An incremental analysis will be conducted for the women who had prior pregnancies. A full plan is currently under discussion.

## Discussion

The rate of congenital malformations in this district (6.8–12.4%) [1] is higher than that of the background population (1.7%, from 2005 to 2010) [28], as well as the rates reported by two previous Australian studies conducted in Melbourne (4%) [29] and Adelaide (5%) [30] and several international studies from England, Wales, and Northern Ireland (4.6%) [31] and from north west England (9.4%) [32]. Similarly, the rate of preeclampsia is considerably higher in our study than that reported from Melbourne (12.2% vs. 5.0%). However, the rates of macrosomia (34.7%) and caesarean section (57.2%) are slightly lower in our study than those reported by the study done in Melbourne (44% and 62%, respectively) [28]. Our results showed few women (2.4%) with T1D were receiving continuous subcutaneous insulin infusions (CSII) in comparison with those in the United Kingdom (20%) [33, 34], which could be due to its out of pocket cost in Australia and HCP time requirements [35].A range of barriers have been identified, similar to those reported previously [36], that are likely to have contributed to limitations in clinical care and self-management. These in turn will have increased the likelihood of unplanned pregnancies and poor pregnancy outcomes (e.g. congenital malformations) [36, 37]. In view of the high rate of congenital malformations, and existing barriers to optimal care, the development and implementation of a district-wide contraception and pre-pregnancy program was considered to be an urgent initiative. The program does not address issues related to undiagnosed diabetes and its association with adverse pregnancy outcomes.

The DCAPP program is the first Australian diabetes pre-pregnancy intervention program based upon various research tools (comprehensive literature review, audits, interview surveys and in-depth focus-groups and interviews in addition to multidisciplinary meetings with HCPs). It has targeted women’s partners who can potentially influence women’s PPM and contraceptive uptake decision making [38]. Furthermore, the program is based upon principles of integrated care, with a primary-secondary care partnership, including aspects of clinical care within primary care, rather than simple specialist/hospital educational interventions. The program has been developed to allow both sustainability and scalability. In the few places where such a comprehensive program has been put into place, malformations in particular were (cardiac, spinal) has been estimated to have a lifelong cost of $1,000,000 [39].

As a program across a population of almost a million [40], over 450 general practices, 188 pharmacies and five hospitals with birthing facilities, DCAPP is faced with a range of challenges associated with large scale programs. As a result, the program will be implemented practice by practice, clinic by clinic and pharmacy by pharmacy with the support of other organisations including the Primary Health Network that supports all general practices across SWS. By including both general practices and pharmacies in the program, we expect to also reach those women with T1D/T2D who attend private providers (e.g. endocrinologists, educators) for their care. The degree of participation by those under the public and private sectors (and their pregnancy outcomes) will be included in the evaluation.

With such an extensive range of providers, the role of the coordinator, the DCAPP team and the within clinic/practice contacts will also be crucial in identifying those reached or otherwise by the roll out and in developing new strategies to reach women who are hard to reach. For example, women with T2D on diet management alone might not attend pharmacies and may need additional practice based strategies. Evaluation of the uptake of the clinic/practice contact and their reporting will be important to identify the need for any further support/incentives for this role.

SWS is characterised by its rich cultural diversity (represented by residents from East and South Asia, the Middle East and the Pacific Islands) [40]. Of particular interest, as the program is rolled out, will be the uptake of contraception among cultural groups who may have religious objections to pharmacological or barrier contraception methods. This is likely to require additional work (e.g. a targeted optimisation program, including avoidance of potentially teratogenic agents) among women with T2D not taking contraception.

Myths and misconceptions as well as lack of knowledge of emergency contraception, and their male partners’ perspectives regarding contraception, are the most common barriers to utilising contraception for women (especially in those with lower levels of education) [41–44]. Previous studies [44, 45] have also highlighted the important role of social media and group-session workshops in raising and updating women’s knowledge of contraception uptake. Methods to achieve this are included as part of the DCAPP.

Our chart reviews and interviews/focus groups were limited due to small sample size/number of participants, leading to wide limits for the pregnancy outcomes and possible under-reporting the participants’ comments and suggestions. Nevertheless, our results have shown the severity of poor pregnancy outcomes in women living in SWS, emphasising the need for an intervention program in this district.

## Conclusions

With the high rates of congenital malformations and high ethnic diversity in this district of Sydney, it is hoped that this program will sustainably reduce adverse pregnancy outcomes in women with pre-existing T1D/T2D. DCAPP is based upon formative research, current best practice, a partnership across primary and secondary care, with new facets including social media and information for partners that have not been included in previous PPM programs. The role of implementation, outcome and cost-effectiveness monitoring will be crucial to assess whether the program should be continued and extended to other areas.

## Additional file

Additional file 1: Example excerpts reflecting the themes identfied. Perspectives of women and their partners on the barriers of pre-pregnancy management and contraception uptake and the importance of having access to additional educational resources. (DOCX 33 kb)

## Abbreviations

BMI: Body mass index
DCAPP: Diabetes Contraception and Pre-pregnancy Program
EASIPOD: East Anglia Study group for Improving Pregnancy Outcomes in women with Diabetes
GP: General practitioner
HCP: Health care professional
PPC: pre-Pregnancy Care
PPM: Pre-Pregnancy Management
SWS: South Western Sydney
T1D: Type 1 diabetes
T2D: Type 2 diabetes
