# Supplementary material for: Development of an integrated, district-wide approach to pre-pregnancy management for women with pre-existing diabetes in a multi-ethnic population
Source: BMC Pregnancy Childbirth. 2018 Oct 15;18:402. doi: 10.1186/s12884-018-2028-2 (PMC6190660; doi:10.1186/s12884-018-2028-2)
Supplement: Supplementary file 1 — Example excerpts reflecting the themes identfied. Perspectives of women and their partners on the barriers of pre-pregnancy management and contraception uptake and the importance of having access to additional educational resources. (DOCX 33 kb) [file 12884_2018_2028_MOESM1_ESM.docx]

**Additional file 1:** Example excerpts reflecting the themes identified

| **Participants** | **Theme** | **Example excerpt(s)** |
| --- | --- | --- |
| **Patients** | Early referrals for longer consultations | “*dietitians from the clinic [could] call patient[s] for follow-up on diet management*” (W107-T2D).  *“the nurses from the diabetes clinic used to call in and check my sugars, advise me to eat this [or] that, so it was very helpful*” (W106-T2D).  *:pre-pregnancy education on glucose control and the importance of HTN medication cessation’* (W101-T2D) |
|  | Barriers to contraception and PPM | “*It was a two-hour talk…I actually found it was just too overwhelming and it did not make sense to me because I was not yet pregnant. So hearing all these things about complications and risks you are just going “yep I’ve been told that my whole life”, you didn’t hear it,”* (W116-T1D).  “*you can lead a horse to water but you can’t make it drink…it’s up to them whether they want to take the information on board or they just want to shrug it off and go ‘I don’t care’*” (W101-T2D). |
|  | Importance of awareness and access to educational resources | “*Facebook pages are great…if it wasn’t for that I wouldn’t know…so I found it really helpful*” (W122-T2D).  “*I said that the doctor told me to reduce weight. [The GP] said if you wait for your weight loss then it will take too much. They [friends] said don’t worry about that, there are more heavier persons than you who become pregnant, so I think [later is] a better time to start”* (W117-T2D). |
| **Partners** | Partner support and investment in engagement | “*I think if, we really want information and we look for it we are getting it so, I think it’s fine, but some more leaflets at the GP, we find a lot of brochures at the GP place so if there were some are more specifically for pre-pregnancy and diabetic-complicate like that, if those things are highlighted that would be handier*.” “*Educational courses would be awesome if it is possible”* (M123-T2D). |
